# Enhancing the Diagnosis of Behçet’s Disease Using Machine Learning: A Comparative Study on Clinical Data From Saudi Arabia

**DOI:** 10.1155/ijta/6157852

**Published:** 2026-01-24

**Authors:** Hanady Alalwany, Nofe Alganmi, Yasser Bawazir, Mohammad Mustafa, Heba Abusamra, Haneen Banjar, Areej Alhothali, Somayah Albaradei

**Affiliations:** ^1^ Department of Computer Science, Faculty of Computing and Information Technology, King Abdulaziz University, Jeddah, Saudi Arabia, kau.edu.sa; ^2^ Center of Excellence in Genomic Medicine Research, King Abdulaziz University, Jeddah, Saudi Arabia, kau.edu.sa; ^3^ Centre of Artificial Intelligence in Precision Medicines, King Abdulaziz University, Jeddah, Saudi Arabia, kau.edu.sa; ^4^ Department of Medicine, Faculty of Medicine, King Abdulaziz University, Jeddah, Saudi Arabia, kau.edu.sa; ^5^ Department of Medicine, University of Jeddah, Jeddah, Saudi Arabia, uj.edu.sa

**Keywords:** Behçet’s disease (BD), clinical features, diagnosis of diseases, explainable artificial intelligence (XAI), machine learning (ML), SHAP analysis

## Abstract

Behçet’s disease (BD) is one of the most difficult diseases to diagnose in the field of rheumatic immune diseases because it is rare, has many different symptoms, and we do not know much about how it works. Instead of trying to make a direct clinical diagnosis, this study was set up as an exploratory investigation to find out more about BD and figure out which clinical and laboratory features are most important. To accomplish this, clinical data were gathered from 148 patients (76 with bipolar disorder and 72 with rheumatoid arthritis) at the Rheumatology Clinic of King Abdulaziz University. We used several machine learning (ML) algorithms, such as decision tree, bagging, random forest (RF), XGBoost, and support vector machines (SVMs), to see if they could learn patterns that set BD apart from other rheumatic diseases. We used three different methods to find out how important each feature was: built‐in model importance, permutation‐based analysis, and Shapley additive explanation (SHAP) values. The ML models worked well, with the RF getting the best accuracy (96.7%) and an area under the curve (AUC) of 1.0. XGBoost came in second with an AUC of 0.9985. The feature analysis showed that the results were partially in line with established diagnostic criteria (Japan, ISG, and ICBD), with oral ulcers being the most important feature. Overall, this study serves as an exploratory framework to deepen understanding of BD’s distinctive characteristics and underlying feature interactions, offering insights that can inform future diagnostic support systems rather than serving as a diagnostic tool itself.

## 1. Introduction

Since Hulusi Behçet first identified BD (Behçet’s disease) in 1937, no definitive diagnostic test or single specific symptoms have been established for the condition. Diagnosis relies on a clinical approach, where healthcare professionals consider a combination of clinical features and patient medical history and exclude other potential conditions. The typical clinical features include recurrent, painful oral and genital ulcers, with inflammation that can impact the eyes, skin, blood vessels, joints, and central nervous system [1]. The phenotypic expression of BD is complex and varies across different genetic and environmental factors [2]. Diagnostic criteria can be categorized into different phenotypes. For example, eye disease may present as either anterior or posterior uveitis; skin manifestations might include pustulosis, erythema nodosum, or superficial venous thrombosis; and vascular involvement can range from aneurysms to embolisms. Furthermore, patients may exhibit a heterogeneous range of symptoms, from specific symptoms to unpredictable relapses that lead to severe clinical flares that can lead to organ damage or even death [3]. Diagnosing BD has become increasingly complex, leading to the development of more diagnostic criteria than for any other medical condition. These criteria include Curth (1946), Hewitt (1969), Mason (1971), Japan (1972), Hubault (1974), O’Duffy (1974), Cheng (1980), Dilsen (1986), Japan revised criteria (1988), the International Study Group on Behçet’s Disease (ISG criteria, 1990), Iran traditional criteria (1993), Iran Classification Tree (1993), Dilsen revised criteria (2000), Korea Criteria (2003), the International Criteria for Behçet’s Disease (ICBD, 2006), and the revised ICBD (2010) [4, 5].

Among the key diagnostic criteria developed for BD, the Japanese criteria were established by the Japan Research Committee for Behçet’s Disease in 1972. This diagnosis is based on a set of symptoms, including recurrent aphthous oral ulcers, skin lesions, ocular inflammation, and genital ulcers, all classified as “major symptoms.” Patients exhibiting all four major symptoms throughout their clinical course are defined as having complete‐type BD. These criteria were revised in 1988 to include five additional minor findings: arthritis, intestinal ulcers, epididymitis, vascular lesions, and neurological disease. If an ocular lesion is present, only one additional major finding is required for diagnosis. In the absence of ocular findings, the presence of the other three major findings is necessary for diagnosis [4, 6].

The ISG criteria are considered more stringent than the Japanese criteria; they require the presence of oral ulceration along with any two of the following for a BD diagnosis: genital ulceration, specified eye lesions, or specified skin lesions. Additionally, a pathergy test is included in the criteria, which is rarely positive in Japanese BD patients [7].

Although the ISG criteria demonstrated notable specificity, they exhibited limited sensitivity, failing to identify a significant subset of patients with BD [8]. The ICBD criteria were developed to address the ISG’s lack of sensitivity. In the ICBD criteria, different features are assigned specific scores: ocular lesions, oral aphthosis, and genital aphthosis each receive 2 points, while skin lesions, central nervous system involvement, and vascular manifestations are assigned 1 point each. The pathergy test, if used, is also assigned 1 point. A patient with a total score of 4 or more points is classified as having BD [9]. Machine learning (ML), as a data analysis methodology, constructs algorithms through iterative data learning. It has demonstrated significant potential in improving patient diagnoses, forecasting outcomes, and evaluating treatment responses [10].

In recent years, there has been a growing interest in the application of ML techniques in the field of rheumatology, particularly for improving the accuracy and speed of diagnosing complex autoimmune diseases. Published studies have demonstrated the potential of ML models in distinguishing between overlapping clinical presentations, optimizing feature selection, and enhancing diagnostic workflows for diseases such as systemic lupus erythematosus (SLE) and rheumatoid arthritis (RA). These advancements support the relevance of our current study and emphasize the importance of integrating AI tools into clinical decision‐making processes in rheumatology [11, 12].

ML in medicine should not be viewed merely as a diagnostic replacement but rather as a framework for knowledge discovery that enhances clinicians’ understanding of disease complexity and supports data‐driven reasoning [13].

Additionally, challenges in diagnosing BD underscore the importance of utilizing modern technologies, such as ML, to aid in early diagnosis. However, there is a limited amount of published research employing artificial intelligence or ML techniques specifically for diagnosing BD [14, 15]. Moreover, those studies that did use these technologies often focused on specific aspects of the disease rather than comprehensive diagnosis. According to the new vision [16], this study embraces the idea of high‐performance medicine, in which artificial intelligence and human expertise combine to enhance clinical judgment and uncover new insights rather than automate diagnosis. Therefore, the current study does not seek to duplicate or substitute the clinical diagnostic procedure. Rather, it employs an exploratory methodology to extract significant insights from clinical data, comprehending the interplay between various clinical and laboratory variables and identifying the characteristics that most strongly define BD.

This is consistent with the new paradigm of “knowledge discovery in medical data,” in which ML is used as a framework to comprehend feature interactions and disease mechanisms rather than as a diagnostic tool. In order to promote transparency and knowledge generation rather than just prediction, explainable AI techniques like SHAP allow researchers to shift from local prediction explanations toward a global understanding of how features contribute to clinical outcomes [17].

This research is aimed at addressing this gap by evaluating the effectiveness of various ML models and the use of different techniques to explain the importance of features in contributing to understanding the features most associated with the disease. Additionally, this research will contribute to the creation of new local clinical datasets for BD patients. To our knowledge, this is the first primary database collected within the Kingdom of Saudi Arabia for this purpose.

## 2. Main Contributions

• Create a local dataset that includes clinical data on both BD patients and patients with other rheumatic diseases before diagnosis. This dataset will be the first of its kind in the region. Considering the variations in disease patterns influenced by environmental factors, this dataset could prove to be highly valuable.
• Evaluate the effectiveness of various ML models in diagnosing BD by applying them to the new dataset and using various performance metrics for evaluation.
• Use different explainable artificial intelligence (XAI) techniques to elucidate the importance of features, identifying the factors most associated with the diagnosis of BD. This contributes to supporting doctors and enabling the rapid diagnosis of patients.

## 3. Literature Review

Below, we present a review of the literature regarding the use of ML techniques in diagnosing BD. Following this review, Table 1 provides an analytical summary of the key findings. Collectively, these studies indicate that only a few published works have applied machine learning approaches to the diagnosis of BD. Few published studies have identified the use of ML for diagnosing BD. One of the most significant research papers, which achieved up to 99.64% accuracy in disease prediction, highlights the use of ML techniques to identify genetic markers for BD. The researchers applied various ML algorithms to analyze genetic data and identify single‐nucleotide polymorphisms (SNPs) characteristic of the disease. These algorithms enabled the researchers to sift through large datasets and pinpoint specific SNPs that could serve as reliable biomarkers. Beyond diagnostic accuracy, this study represents an example of knowledge discovery, as it deepened the understanding of the genetic background of BD and revealed molecular patterns that may underlie disease susceptibility, consistent with the broader role of ML in uncovering biological insights rather than serving solely as a diagnostic instrument [18]. Another paper explores the use of ML techniques to identify biomarkers associated with BD through plasma proteomic analysis. By employing advanced ML algorithms, the researchers analyzed proteomic data to discover immune‐related biomarkers indicative of the disease. The study’s findings underscore the effectiveness of these MLs in managing complex biological data and identifying significant protein markers. Such approaches contribute to knowledge generation regarding the immune response in BD, allowing researchers to map protein‐level alterations and their potential mechanistic implications, which complements the movement toward explainable and discovery‐oriented AI in medicine [19]. Notably, one study applied a deep learning model to distinguish between colonoscopy images of intestinal BD, Crohn’s disease (CD), and intestinal tuberculosis (ITB) [20]. This work extended ML applications from clinical data to image‐based recognition, illustrating how deep learning can reveal visual and histopathological features that differentiate intestinal BD from phenotypically similar diseases. Such image‐based discovery further supports the role of AI as a tool for understanding disease morphology. Two studies examining clinical features observed in patients have focused on ocular risks and factors strongly linked to the development of ocular complications in BD. In the first study, multinomial logistic regression was used to determine the misclassification rate of BD‐uveitis among 1012 panuveitis cases, including 194 cases of BD with uveitis. The overall accuracy achieved was 96.3% in the training set and 94.0% in the validation set [21]. In the second study, a cohort of 1049 subjects from the Egyptian College of Rheumatology, encompassing 26 features, was used. ML models were applied to predict risk factors for vision‐threatening BD (VTBD) in patients. The study also examined the clinical significance and direction of each factor using SHAP values. The results identified higher disease activity, thrombocytosis, a history of smoking, and daily steroid dosage as the key factors associated with VTBD, achieving a notable 95% accuracy in the test set [22]. Additionally, the use of SHAP analysis in this study exemplifies how interpretable ML methods can provide transparent explanations of feature contributions, thereby transforming traditional prediction into knowledge discovery about disease mechanisms. Previous studies have demonstrated that ML techniques possess strong discriminatory capacity for the diagnosis of BD. This finding highlights the potential for applying these techniques to various aspects of the symptoms and complications associated with BD. When considered collectively, these studies demonstrate how ML enhances diagnostic precision while also revealing biological, proteomic, and clinical patterns that deepen our understanding of the pathophysiology of the disease. Even though these studies offer insightful information about the impact of BD, their generalizability to other disease manifestations is limited because they concentrated on particular aspects of the condition. Their shortcomings highlight the need for more studies that fully examine the dangers and other elements that are strongly associated with the emergence of complications in BD patients. To further enhance diagnostic precision and patient outcomes, ML applications in BD diagnosis must be investigated beyond particular subsets or complications. By taking a more comprehensive, discovery‐oriented approach, we hope to enhance these earlier studies in this work. In order to find and analyze the most informative characteristics linked to BD, our study applies multiple ML models to clinical data rather than concentrating on a single dimension like genetics, proteomics, or ocular complications. This study combines diagnostic modeling with explainable AI to highlight important disease features using three different approaches: model‐based feature importance, permutation‐based analysis, and Shapley additive explanation values. In addition to supporting the new paradigm of applying ML for knowledge exploration and insight generation rather than just classification, this integration allows for a deeper understanding of the clinical profile of BD.

**Table 1:** Studies that applied machine learning with Behçet’s disease.

**Study ref**	**Data**	**Method name**	**Contributions**	**Strengths**	**Limitations**	**Year**
16	1215 BD, 1278 control	‐ ML models (LR, SVM, RF, k‐NN, XGBoost, voting ensemble algorithms) ‐ Feature selection methods	Identifying SNP genotyped sets for BD diagnosis	‐ Best accuracy (99.64%) with LR ‐ Detailed feature selection process	Focused on genetic data	2022
17	27 BD, 25 control	‐ ML models (NB, SVM, XGBoost, RF, and NN)	Identify immune response‐related proteins for BD	‐ Best accuracy (75.0) with RF	Focused on proteomics	2023
18	2852 BD 2123 CD 1642 ITB	‐ DL (CNN) applied on imaging	Real‐time differentiation between similar diseases	‐ Accuracy (85.62%) ‐ Provides a practical tool	Focused on imaging Intestinal Behçet’s	2021
19	1012	‐ LR model	Classification criteria for Behçet disease uveitis	‐ Accuracy (94.0%)	Focused on ocular risks	2021
20	1049	‐ ML models (XGBoost, RF, extra tree, SVM, ANN, MLP, and LR) ‐ SHAP	Machine learning for classifying (VTBD)	‐ Best accuracy (95.0%) with XGBoost ‐ Examined factors associated with VTBD	Focused on ocular risks	2023

## 4. Materials and Methods

**Figure 1:**
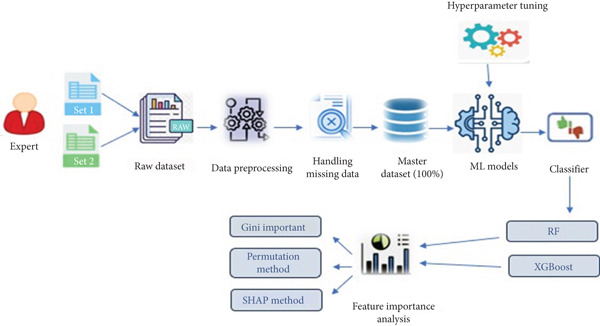
Study design workflow for data management and BD prediction model development.

The methodology design of this study encompasses a comprehensive framework aimed at developing a predictive model for BD. The framework comprises four primary components: data gathering and dataset construction, data preprocessing, ML model development and evaluation, and feature importance, as illustrated in Figure 1.

### 4.1. Data Collection

The data for this study were obtained through a comprehensive analysis of medical records from patients diagnosed with BD and RA at the Rheumatology Clinic of King Abdulaziz University Hospital (KAUH) in Jeddah, Saudi Arabia. The study was conducted under ethical standards, having received approval from the Ethics Committee at KAUH under IRB No. HA‐02‐J‐008. Informed consent was obtained from all participants before data collection. Two distinct datasets were initially compiled for this study. The first dataset is raw data for Behçet’s patients. This dataset comprises detailed information on patients diagnosed with BD, including personal and demographic data, clinical symptoms, laboratory test results, and clinical diagnosis. The statistical details of the raw dataset features are provided in Table S2, while the frequency distributions and missing data summary are reported in Table S4. The clinical symptoms recorded include oral and genital ulcers, ocular and skin lesions, neurological manifestations, arthritis, erythema nodosum, superficial phlebitis, deep vein thrombosis, large vein or arterial thrombosis, retinal vasculitis, and a positive pathergy test. The second dataset is raw data for RA patients. This dataset includes comprehensive personal information, demographic data, a set of laboratory test results, and clinical diagnoses. After the data preprocessing operations, which we will discuss in detail in the next section, both datasets were integrated into a single, comprehensive dataset as detailed in Table 2. This integrated dataset includes demographic variables, age categories (< 20 years, 21–30 years, and > 30 years), and nationality (categorized as Saudi Arabian [SA] or non‐Saudi Arabian [non‐SA]). Clinical symptoms include oral and genital ulcers, ocular and skin lesions, neurological manifestations, arthritis, erythema nodosum, superficial phlebitis, deep vein thrombosis, large vein or arterial thrombosis, retinal vasculitis, and a positive pathergy test. Laboratory test results include erythrocyte sedimentation rate (ESR), C‐reactive protein (CRP), hemoglobin (Hb), platelet count (PLT), white blood cell (WBC) count, aspartate aminotransferase (AST), alanine aminotransferase (ALT), bilirubin, albumin, rheumatoid factor, anticyclic citrullinated peptide (anti‐CCP), partial thromboplastin time (PTT), prothrombin time (PT), international normalized ratio (INR), creatinine, antinuclear antibodies (ANAs), anti‐DNA antibodies, anti‐Sjögren’s syndrome A antibodies (anti‐SSAs), scleroderma antibodies (SCL‐70), antineutrophil cytoplasmic antibodies (ANCAs), anticardiolipin antibodies, and viral markers for hepatitis C (HCV) and hepatitis B (HBV). The final diagnosis categorizes patients as Behçet or non‐Behçet. The study cohort comprised 148 participants, divided into two groups: 76 patients diagnosed with BD and 72 patients diagnosed with RA. The term “non‐Behçet” refers to the RA group, as they were not considered healthy controls. Data were collected during the diagnostic phase, before the initiation of any treatment. The similarity in size and characteristics between the two groups suggests a balanced dataset, making it suitable for comparative analysis.

**Table 2:** List of features: Demographic, clinical characteristics, and laboratory tests.

**Attribute name**	**Type**	**Coding**			**Category**
Age	Categorical	1 (≤ 20)	2 (21–30)	3 (> 30)	Demographic
Nationality	Categorical	1 (SA)	0 (not SA)		Demographic
Oral ulcers	Categorical	1 (yes)	0 (no)		Symptom
Genital ulcers	Categorical	1 (yes)	0 (no)		Symptom
Ocular lesions	Categorical	1 (yes)	0 (no)		Symptom
Skin lesions	Categorical	1 (yes)	0 (no)		Symptom
Neurological manifestations	Categorical	1 (yes)	0 (no)		Symptom
Arthritis	Categorical	1 (yes)	0 (no)		Symptom
Erythema nodosum	Categorical	1 (yes)	0 (no)		Symptom
Superficial phlebitis	Categorical	1 (yes)	0 (no)		Symptom
DVT	Categorical	1 (yes)	0 (no)		Symptom
Large vein thrombosis	Categorical	1 (yes)	0 (no)		Symptom
Arterial thrombosis	Categorical	1 (yes)	0 (no)		Symptom
Uveitis	Categorical	1 (yes)	0 (no)		Symptom
Retinal vasculitis	Categorical	1 (yes)	0 (no)		Symptom
Positive pathergy test	Categorical	1 (yes)	0 (no)		Symptom
ESR	Numeric				Laboratory test
CRP	Numeric				Laboratory test
HB	Numeric				Laboratory test
PLT	Numeric				Laboratory test
WBC	Numeric				Laboratory test
AST	Numeric				Laboratory test
ALT	Numeric				Laboratory test
Bilirubin	Numeric				Laboratory test
Albumin	Numeric				Laboratory test
Rheumatoid factor	Categorical	1 (negative)	0 (positive)		Laboratory test
Anti‐CCP	Categorical	1 (negative)	0 (positive)		Laboratory test
PTT	Categorical	1 (normal)	0 (abnormal)		Laboratory test
PT	Categorical	1 (normal)	0 (abnormal)		Laboratory test
INR	Categorical	1 (normal)	0 (abnormal)		Laboratory test
Creatinine	Numeric				Laboratory test
ANA	Categorical	1 (positive)	0 (negative)		Laboratory test
Anti‐DNA	Categorical	1 (negative)	0 (positive)		Laboratory test
Anti‐SSA	Categorical	1 (negative)	0 (positive)		Laboratory test
SCL‐70	Categorical	1 (negative)	0 (positive)		Laboratory test
ANCA	Categorical	1 (negative)	0 (positive)		Laboratory test
Anticardiolipin	Categorical	1 (negative)	0 (positive)		Laboratory test
HCV	Categorical	1 (negative)	0 (positive)		Laboratory test
HBV	Categorical	1 (negative)	0 (positive)		Laboratory test
DAG	Categorical	1 (Behçet’s)	0 (not Behçet’s)		Result

### 4.2. Data Preprocessing

Relevant clinical features were carefully selected to create a unified dataset, guided by clinical expertise, literature review, and data availability. Nonessential information, such as patient identification, was removed to focus on diagnostic and clinical variables. Similar columns from two datasets were combined into a single dataset. The similar columns include the following clinical variables: demographic information (age and nationality), diagnostic indicators, and laboratory parameters such as ESR, CRP, Hb, platelets, WBC count, liver enzymes (AST and ALT), bilirubin, albumin, LDL, hemoglobin A1c (HBA1C), and creatinine. It also includes autoantibodies and immune markers (ANA, anti‐DNA, anti‐SSA, ANCA, SCL‐70, and anticardiolipin) and other clinical measures (ECHO EF%, HCV, and HBV status). Then, columns containing numerical and textual data were standardized and transformed into categorical variables to ensure consistency and comparability, particularly for anti‐DNA, ANA, PTT, PT, INR, and LDL markers. These transformations were applied according to standard laboratory measurement ranges, and the complete value mapping and categorical encodings are detailed in Table S3. Symptom columns for an RA group were populated based on physician‐documented data from the corresponding diagnostic category. For example, symptoms specific to BD were recorded as “no” for patients diagnosed with RA, reflecting the clinical evaluations captured in the source data. Similarly, laboratory tests such as rheumatoid factor and anti‐CCP were positive in RA patients and negative in BD patients, as observed in the clinical dataset. All columns were then encoded with categorical variables to zero and one and numerical variables to ranges. Upon completing these procedures, 43 features were extracted. This integration resulted in a comprehensive dataset suitable for analyzing clinical outcomes and enhancing diagnostic accuracy. However, the issue of missing data remains a challenge, which will be addressed in detail in the following section.

### 4.3. Handling Missing Data

Clinical datasets commonly contain three types of missing data: missing completely at random (MCAR), missing at random (MAR), and missing not at random (MNAR). Removing patient records can undesirably reduce the dataset size and eliminate potentially informative patterns. Therefore, we quantified the extent of missingness for each feature, as reported in Table S4 and applied a cutoff threshold of 25% for feature inclusion. Consequently, features such as echocardiogram for ejection fraction (ECHO EF)%, HBA1C, and cholesterol blood (LDL) were excluded, and the remaining missing values were addressed through imputation rather than removing patients with incomplete records. Multiple imputation techniques were employed using the “impute” command in SPSS, utilizing the fully conditional specification method for categorical variables and the predictive mean matching method for continuous variables, with five imputations performed. Subsequently, the imputed data were consolidated into a single, complete dataset using the output management system (OMS) in SPSS. Following this phase, 40 features were extracted, as outlined in Table 2.

### 4.4. ML Model Development

By evaluating the viability and potential of ML techniques in aiding the diagnosis of BD, the current study is aimed at exploring and improving the current understanding of BD rather than just using AI to mimic the decision‐making process of seasoned clinicians. The purpose of this exploratory study was to assess how ML‐based methods can help doctors spot unique clinical patterns and aid in the diagnostic process. To achieve this objective, ML models were employed instead of deep learning methods, as the dataset lacked image‐based data and was relatively small in size. Given that the dataset was labeled into two classes (BD and non‐BD), the problem was formulated as a supervised ML task. After comprehensive preprocessing, a set of classification algorithms—including decision tree (DT), bagging, random forest (RF), XGBoost, and support vector machine (SVM)—were implemented to perform binary classification and distinguish BD from non‐BD cases. These models were selected due to their frequent use in medical research and their proven effectiveness in handling structured clinical data.

The dataset, consisting of 148 patient records, was divided into training (80%) and testing (20%) subsets, comprising 118 and 30 records, respectively. Model performance was optimized using the GridSearchCV procedure, which systematically explores combinations of hyperparameters to identify the optimal configuration through cross‐validation. Specifically, a fivefold cross‐validation strategy was applied to the training set, subdivided into training and validation portions to prevent data leakage and ensure model robustness. The final evaluation was performed using the independent testing set. All models were implemented in Python using the scikit‐learn library.

### 4.5. Model Evaluation Metrics

In order to evaluate and identify the most accurate model, the analysis employed the computation of a confusion matrix for each model. This matrix encompasses essential metrics, including true positive (TP), true negative (TN), false positive (FP), and false negative (FN), with BD categorized as the positive group and non‐BD as the negative group. Additionally, it calculated performance measures such as true positive rate (TPR), true negative rate (TNR), accuracy, sensitivity, specificity, F‐score, and AUC, utilizing the formulas outlined in Table 3. The utilization of a confusion matrix facilitated a direct comparison of each model’s performance on the testing dataset, ensuring a comprehensive assessment of their predictive capabilities.

**Table 3:** Formulas and descriptions of the performance evaluation metrics.

**Metrics**	**Description**
Accuracy	The accuracy metric measures the ratio of correct predictions (BD and non‐BD) over the total number of instances evaluated.
AUC	This metric is used to measure the ability of a binary classifier to distinguish between BD and non‐BD.
Sensitivity (TPR)	This metric is used to measure the fraction of positive group (BD) that are correctly classified.
Specificity (TNR)	This metric is used to measure the fraction of negative group (non‐BD) that are correctly classified.
F1‐score	This metric is used to measure the accuracy of the test.

### 4.6. Feature Importance

To discern highly essential features crucial for distinguishing between BD patients and non‐BD individuals, three distinct methodologies were employed to ascertain feature importance derived from both RF and XGBoost models: (i) Built‐in feature importance: This method involves utilizing the inherent feature importance functionality within the RF and XGBoost models. This technique quantifies the significance of each feature based on its contribution to the model’s predictive performance. (ii) Permutation‐based feature importance: This approach utilizes a technique aimed at estimating the importance of each feature by assessing the impact of shuffling the feature values on the model’s predictive accuracy. Specifically, the permutation feature importance algorithm measures the increase in prediction error when a particular feature is omitted during permutation, thereby identifying features crucial for accurate predictions. Implementation of this technique was achieved using the “permutation‐importance” function from the “scikit‐learn” package in Python. (iii) SHAP value‐based feature importance: Utilizing SHAP values, a game theory–inspired approach to feature analysis, this methodology delves into how an ML model forecasts the target variable. SHAP values reveal insights into the model’s prediction process by measuring the average absolute value of SHAP across features. Following this, a stacked bar plot is crafted to illustrate the significance of each feature in influencing the model’s predictions [23].

Additionally, a preliminary evaluation of the relationship between clinical characteristics and disease diagnosis was performed using a chi‐square test, and the complete results are presented in Table S5. The ML‐based feature importance, which offers more profound insights into the models’ predictive behavior and the clinical significance of each feature, was the study’s main focus. Together, these methodological steps sought to determine whether ML models could facilitate the diagnostic process of BD through data‐driven exploration and whether they could reveal clinically meaningful insights.

## 5. Results

### 5.1. Performance of Various Prediction Models

In this section, we present five modeling experiments—DT, bagging, RF, XGBoost, and SVM—conducted on the same stratified clinical dataset to diagnose BD. Each model was tuned via fivefold cross‐validation (optimizing ROC‐AUC), evaluated on an 80/20 train‐test split, and assessed with consistent metrics. We describe the experiment in prose and reference the illustrative consolidated comparison table.

Table 4 summarizes the comparative performance of the five optimized ML models based on their best parameter combinations obtained through GridSearchCV. Overall, the RF, XGBoost, and SVM models achieved the highest accuracy rates of 96.7% (97%), while the DT and bagging classifiers followed with 93.3%. In terms of AUC, the RF model demonstrated the best discriminative ability with an AUC of 1.000, followed closely by XGBoost with 0.9985. Bagging also achieved a high AUC value of 0.9917, while SVM and DT recorded slightly lower values of 0.9739 and 0.9583, respectively. Regarding sensitivity, both RF and XGBoost achieved perfect recall (100%), followed by bagging (100%) and DT (92.9%), while SVM recorded 92.9%. For specificity, SVM outperformed the other models with 100%, while DT, RF, and XGBoost maintained high values of 93.8%, and bagging showed the lowest at 87.5%. These findings indicate that ensemble methods, particularly RF and XGBoost, provided the most reliable performance for the classification task, with SVM also showing strong generalization ability compared to single classifiers.

**Table 4:** Model’s evaluation results on test sets.

**ML model**	**Best parameter combination**	**Best AUC**	**Accuracy**	**Sensitivity**	**Specificity**	**F1-score**
Decision tree	Criterion: entropy Max_depth: 8 Max_features: sqrt	0.9583	0.9333	0.9286	0.9375	0.93
Bagging	n_estimators: 5	0.9917	0.9333	1.0000	0.8750	0.93
Random forest	Criterion: entropy Max_depth: 4 n_estimators: 50 Max_features: sqrt	1.0000	0.9667	1.0000	0.9375	0.97
XGBoost	Colsample_bytree: 0.6 Gamma: 1.5 Subsample: 1.0 Max_depth: 3 Min_child_weight: 1	0.9985	0.9667	1.0000	0.9375	0.97
SVM	C: 10 Kernel: rbf Gamma: 0.1	0.9739	0.9667	0.9286	1.000	0.97

### 5.2. Error Analysis Using Confusion Matrices

**Figure 2:**
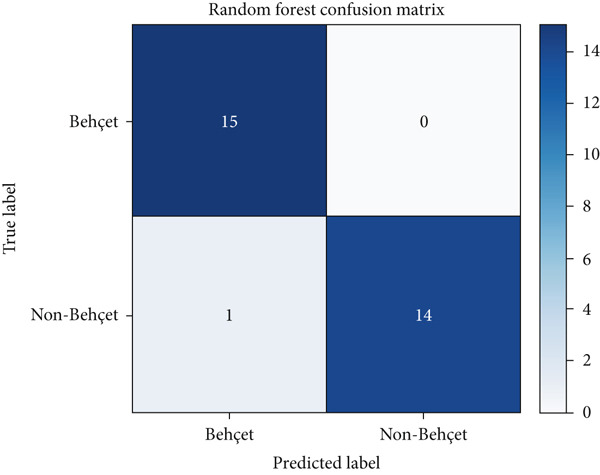
Confusion matrix for the random forest model. The model correctly identified 15 Behçet’s disease (BD) cases and 14 non‐BD cases, with 1 false negative. This demonstrates high sensitivity and specificity, with minimal misclassification.

**Figure 3:**
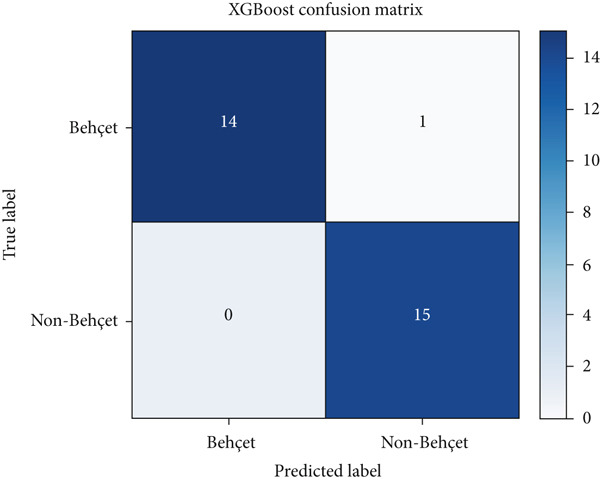
Confusion matrix for the XGBoost model. The model correctly classified 14 BD cases and 15 non‐BD cases, with 1 false positive. The results indicate strong performance with a very low error rate.

To further interpret model behavior, we analyzed the confusion matrices of the two top‐performing models: RF and XGBoost (Figures 2 and 3, respectively).

Both models demonstrated strong predictive performance with minimal misclassifications. The XGBoost model showed one FP and zero FN, while the RF model showed one FN and zero FP. These results indicate a slight trade‐off between sensitivity and specificity across models. Such misclassifications, though few, can have clinical implications, particularly FPs, which may lead to unnecessary treatment, and FNs, which can delay diagnosis. Therefore, even with high‐performance metrics, ML models should be deployed with caution and in conjunction with clinical expertise.

### 5.3. Important Features for Predicting BD Patients

**Figure 4:**
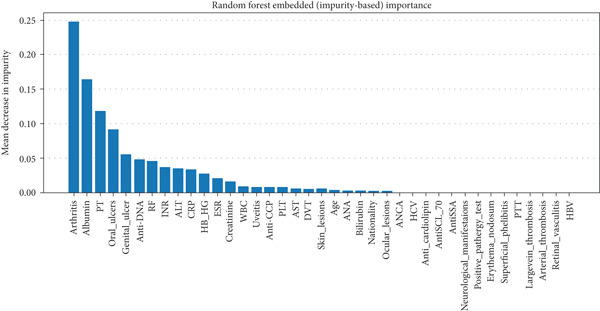
Random forest embedded impurity‐based method (Gini impurity).

**Figure 5:**
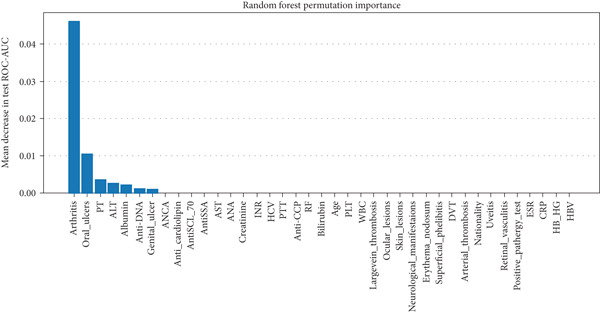
Random forest permutation‐based feature importance method. The top rankings are the most important features, while those bottom rankings matter least.

**Figure 6:**
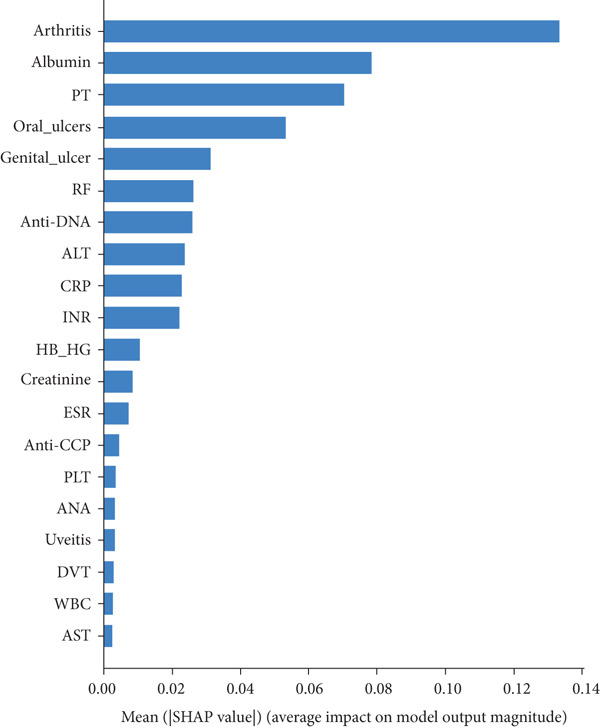
The SHAP feature importance plot; *x*‐axis depicts how individual features’ SHAP values contributed to predicting BD. The features were positioned along the *y*‐axis based on their decreasing importance, where a higher position indicated a higher Shapley value or higher risk of “BD.”

**Figure 7:**
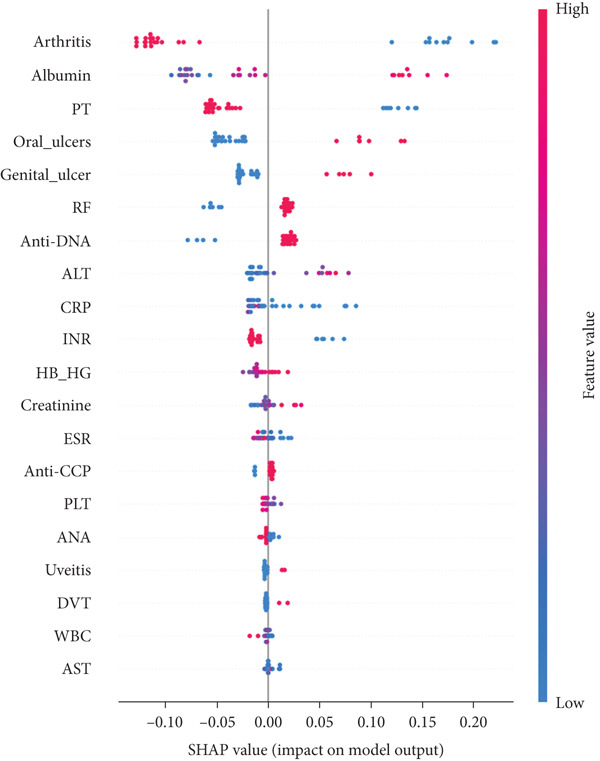
The SHAP summary timelines of the same 10 leading features derived from the RF method. Each point refers to each patient for the respective feature (row).

**Figure 8:**
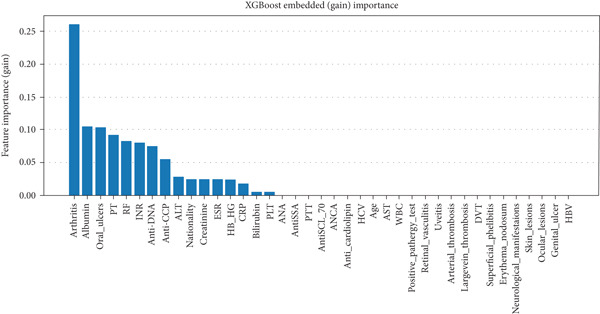
XGBoost features importance (the gain‐based embedded importance).

**Figure 9:**
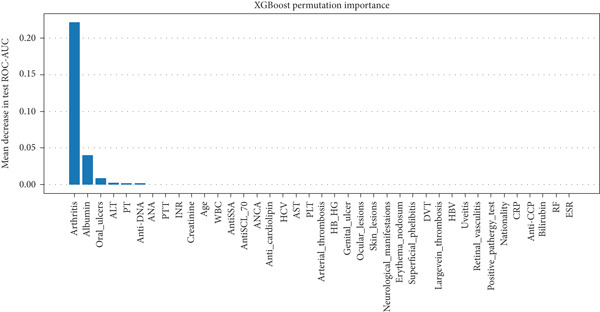
XGBoost permutation‐based feature importance method. The top rankings are the most important features, while those bottom rankings matter least.

**Figure 10:**
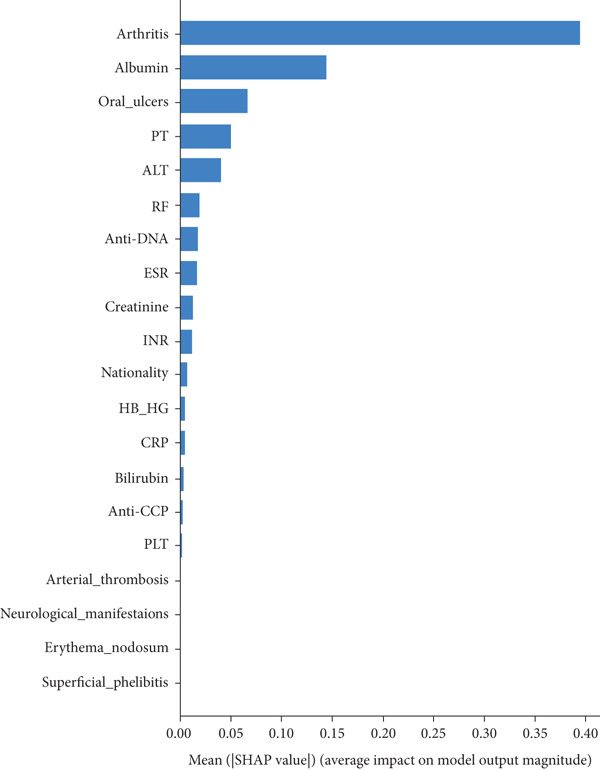
The SHAP feature importance plot shows the average absolute value of SHAP for each feature in a standard bar plot. This plot highlights the Top 10 features obtained from XGBoost, with arthritis as the most influential feature.

**Figure 11:**
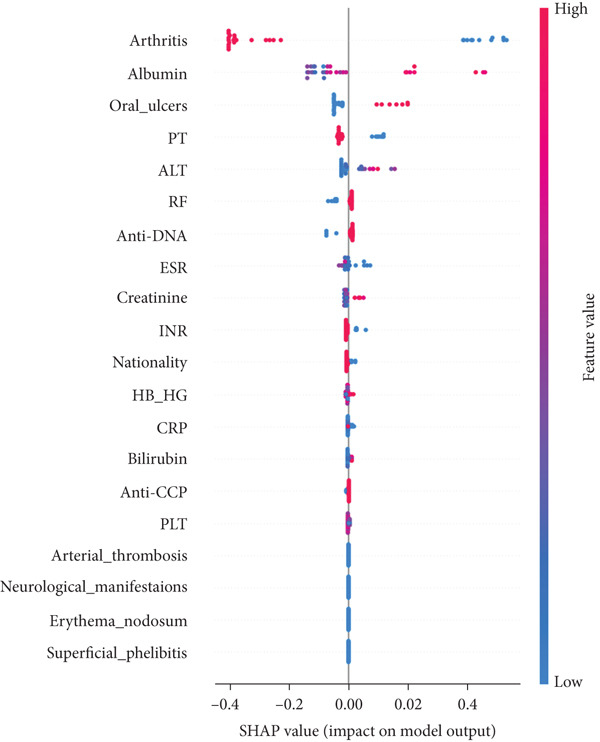
The SHAP summary timelines of the same 10 leading features derived from the XGBoost method. Each point refers to each patient for the respective feature (row).

In RF model (Figures 4, 5, 6, and 7) using the embedded impurity‐based method, arthritis emerged as the most influential feature, followed by albumin, PT, oral ulcers, genital ulcers, and anti‐DNA. These variables consistently reduced impurity across trees, highlighting their strong predictive contribution. In the permutation analysis, permuting arthritis caused the largest decrease in ROC‐AUC, with oral ulcers, PT, ALT, albumin, and anti‐DNA also producing measurable declines, confirming their test‐time stability. The SHAP results reinforced this result, where arthritis exhibited the highest average SHAP value, followed by albumin, PT, oral ulcers, genital ulcers, and anti‐DNA. The beeswarm plots further showed coherent and directional effects of these features on the model’s predictions. In XGBoost (Figures 8, 9, 10, and 11), the gain‐based embedded importance in XGBoost displayed a nearly identical pattern: arthritis held the top rank, with albumin and oral ulcers next, followed by PT and anti‐DNA. The permutation analysis showed that test performance was most sensitive to arthritis and oral ulcers, while albumin, PT, and anti‐DNA also contributed to performance drops. The SHAP analyses confirmed that arthritis had the strongest predictive effect, while albumin, oral ulcers, PT, and anti‐DNA followed with consistent, clinically plausible contributions. Convergence of evidence across both models (RF and XGBoost) and all interpretability methods (embedded, permutation, and SHAP) highlights arthritis, oral ulcers, albumin, and anti‐DNA as the most robust and clinically meaningful features. Arthritis and oral ulcers are hallmark manifestations of BD and are central to international diagnostic criteria. Albumin reflects systemic inflammation and disease activity, making it a strong biochemical marker. Anti‐DNA antibodies, although typically associated with SLE, provided additional discriminative value in separating BD from RA, especially in diagnostically ambiguous cases. Collectively, these four features represent complementary clinical, biochemical, and immunological dimensions, forming a compact yet powerful diagnostic signature. If we were to expand the set of most important features beyond the four basic ones (arthritis, oral ulcers, albumin, and anti‐DNA), the important candidates would be PT, ALT, CRP, creatinine, and ESR. These features are supported by scientific evidence of their association with chronic inflammation, coagulation abnormalities, and organ function, as summarized in Table S1, making them logical additions to enhance the model’s predictive power.

### 5.4. Verification Experience

In order to assess the impact of these key features, we repeat the experiment by testing the five models using the dataset containing only 36 features by eliminating the four identified features of arthritis, oral ulcers, albumin, and anti‐DNA. It became evident that the models exhibited diminished performance. The DT model showed an accuracy of 80%, and the bagging model showed an accuracy of 83% instead of 93%, and also, XGBoost showed an 80% instead of 97%. The RF provided an accuracy of 86%, compared to 97% in the previous experiment with 40 features. The SVM model achieved good accuracy values of 97% and 93%, and the detailed evaluation results are presented in Table 5.

**Table 5:** The model’s evaluation results on test sets, excluding the important features.

**ML model**	**AUC**	**Accuracy**	**Sensitivity**	**Specificity**	**F1-score**
Decision tree	0.8925	0.8000	0.8571	0.7500	0.80
Bagging	0.9451	0.8334	0.9286	0.7500	0.83
Random forest	0.9845	0.8667	0.9286	0.8125	0.87
XGBoost	0.9719	0.8334	0.8571	0.8125	0.83
SVM	0.9141	0.9667	1.0000	0.9375	0.97

## 6. Discussion

BD is primarily diagnosed through clinical assessment rather than laboratory tests. Consequently, various sets of diagnostic criteria have been proposed over the past 50 years to assist medical consultants in identifying BD patients. In this context, the present study was designed as an exploratory and knowledge‐driven investigation to assess the feasibility and potential of ML in facilitating and supporting the diagnostic process of BD. The results obtained and presented in the previous section indicate that ML can serve as a supportive analytical framework by highlighting key predictive features and demonstrating its capacity to assist physicians in improving diagnostic accuracy for BD. Five models were used: DT, Bagging, RF, XGBoost, and SVM. All our models demonstrated good results, with RF and XGBoost showing outstanding performance. The RF achieved an accuracy of 97% and an AUC value of 1, demonstrating its effectiveness and superiority in this study. XGBoost achieved comparable accuracy and an impressive AUC of 0.9985. While this level of performance raises legitimate concerns regarding potential overfitting, several measures were implemented to mitigate such risks. These included fivefold cross‐validation to reduce variance and a dominant trait deletion test to demonstrate the robustness of patterns and consistency across multiple model architectures. Despite the removal of influential features, the models maintained acceptable performance levels, supporting the robustness and stability of the predictive algorithms. Nevertheless, external validation using independent datasets remains essential prior to clinical translation. Various methods were employed to determine the importance of features distinguishing BD patients from non‐BD individuals based on RF and XGBoost classifiers. These included SHAP, impurity‐based importance in RF, and gain‐based importance in XGBoost, which are classified as embedded methods, as well as the permutation method. Features consistently identified across all approaches were regarded as the most crucial predictors (major symptoms), while those appearing in one or more—but not all—methods were considered important yet secondary features. The identification of common features across all approaches highlighted arthritis, followed by oral ulcers, albumin, and anti‐DNA. Additionally, we examined how the remaining features influenced the models’ predictions for BD by conducting an additional experiment that excluded the four key features identified earlier, utilizing only 36 remaining features. The models exhibited poorer performance when arthritis, oral ulcers, albumin, and anti‐DNA were omitted, reinforcing their importance in predictive modeling. The identification of albumin and anti‐DNA as predictive features for BD requires careful clinical interpretation. Although these markers are not classically associated with BD in existing diagnostic criteria, their relevance may reflect underlying inflammatory or autoimmune activity commonly observed in BD patients. Low albumin levels may indicate chronic inflammation or nutritional effects secondary to systemic vasculitis, while anti‐DNA antibodies typically associated with SLE could suggest overlapping immunological pathways or coexisting autoimmune features. These findings were reviewed and validated by a senior rheumatologist consultant (Dr. Yasser Baowzir), who confirmed that such markers, while not disease‐specific, can serve as supportive indicators in the broader clinical context. Further research is warranted to determine whether these markers act as proxies for comorbidities or are integral to BD pathophysiology. Clinically, the findings are noteworthy. By identifying arthritis, oral ulcers, albumin, and anti‐DNA as key diagnostic indicators, the models provide valuable guidance for clinicians evaluating suspected BD cases, particularly in settings with limited access to rheumatology specialists or where clinical presentation is ambiguous. Moreover, the use of interpretable ML techniques (e.g., SHAP) bridges the gap between algorithmic inference and clinical reasoning, fostering trust in AI‐assisted tools. However, these models should be viewed as decision‐support and exploratory analytical frameworks rather than definitive diagnostic systems. Overall, this study does not aim at replacing clinical judgment but at exploring the potential and feasibility of ML in enhancing diagnostic understanding and assisting physicians in identifying meaningful clinical patterns. This comparison demonstrates both overlap and divergence in the clinical markers considered important, highlighting the potential of ML to uncover nuanced and informative patterns relevant to BD diagnosis.

### 6.1. Clinical Implications

Clinically, the study’s results show how a limited number of straightforward, everyday characteristics—like arthritis, oral ulcers, albumin, and anti‐dsDNA antibodies—can help doctors identify BD earlier, especially when the symptoms are vague or overlap. The inclusion of these parameters improves the usefulness of possible ML‐based support tools, as they are frequently utilized in day‐to‐day rheumatology practice. In addition, the use of explainable ML techniques in this situation offers transparency, preventing clinicians from viewing the model as a “black box” and enabling them to comprehend why it generates accurate predictions. This interpretability promotes ethical AI adoption in clinical settings and builds trust. It is important to remember that this is an exploratory, early study. The information comes from a single facility and includes a small sample of people with BD as opposed to RA. These limitations limit generalizability and highlight the necessity of external validation across various populations and hospitals. To ensure the safe, moral, and efficient use of AI‐driven models, responsible clinical implementation will also necessitate strong governance, ongoing monitoring, and cooperation between clinicians and data scientists. These factors draw attention to the advantages and disadvantages of applying ML‐based tools to actual medical decision‐making.

### 6.2. Benchmarking Against Diagnostic Criteria and Feature Interpretation

**Figure 12:**
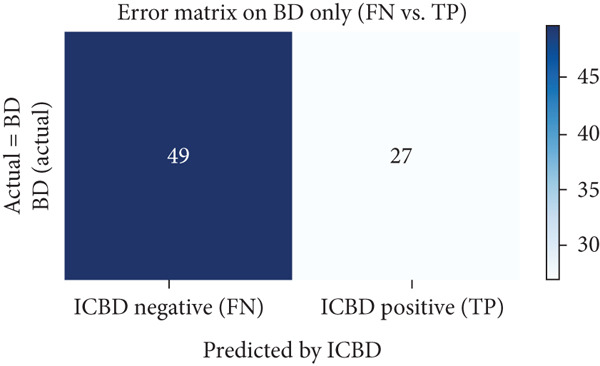
Confusion matrix for Behçet’s disease (BD) patients based on ICBD criteria. Highlighting the limited sensitivity of ICBD in detecting all clinically diagnosed BD patients within this cohort.

To benchmark our findings against established diagnostic frameworks, the ICBD was applied to the same cohort of 76 clinically confirmed BD patients used in the ML experiments. According to the ICBD (2014), the diagnosis of BD is established through a point‐based system in which patients scoring 4 or more points based on clinical manifestations are classified as having BD. The ICBD‐based classification demonstrated limited diagnostic performance, as shown in the confusion matrix (Figure 12), where 49 BD patients were misclassified as non‐BD and only 27 were correctly identified. This result highlights the restricted sensitivity of rule‐based diagnostic systems when applied to a heterogeneous population, such as ours, and underscores the need for complementary data‐driven methods capable of capturing more complex relationships among clinical and laboratory indicators.

Building on this finding, we conducted a detailed comparison between the key predictive features identified by our ML models and the major established diagnostic criteria—namely, the ICBD, ISG, and Japanese frameworks—as summarized in Table 6. The most influential features identified in our study, including oral ulcers, arthritis, anti‐DNA antibodies, and albumin, showed partial alignment with traditional diagnostic systems. The analysis demonstrates a strong overlap in several core manifestations, particularly oral and genital ulcers, which are consistently recognized across all diagnostic frameworks and were among the most influential features in our study.

**Table 6:** A comparison of the BD criteria between this study and published ones. “∗” denotes major symptoms in criteria, “∗∗” denotes included in criteria but not major, and “x” denotes not included in criteria.

**Symptoms**	**Japanese**	**ISG**	**ICBD**	**Our work**
Oral ulcer	^∗∗^	^∗∗^	^∗∗^	^∗∗^
Genital ulcer	^∗∗^	^∗^	^∗∗^	^∗^
Skin region	^∗∗^	^∗^	^∗^	x
Uveitis	^∗∗^	^∗^	^∗∗^	x
Pathergy test	x	^∗^	^∗^	x
Arthritis	^∗^	x	x	^∗∗^
Epididymitis	^∗^	x	x	x
Gastrointestinal	^∗^	x	x	x
Neuro	^∗^	x	^∗^	x
Vascular	^∗^	x	^∗^	x
Albumin	x	x	x	^∗∗^
Anti‐DNA	x	x	x	^∗∗^
PT	x	x	x	^∗^
ALT	x	x	x	^∗^
Creatinine	x	x	x	^∗^
CRP	x	x	x	^∗^
ESR	x	x	x	^∗^

However, arthritis, which emerged as a key predictive feature in our models, does not appear in the ICBD or ISG criteria and is only listed as a minor feature in the Japanese standard—suggesting potential regional or population‐specific variations in disease manifestation. In addition, our models identified albumin and anti‐DNA antibodies as significant laboratory predictors, even though these are not included in any conventional BD criteria. These biomarkers may reflect the systemic inflammatory or autoimmune activity often present in BD, providing novel insights into disease mechanisms. Other laboratory parameters such as PT, ALT, creatinine, CRP, and ESR also demonstrated moderate predictive relevance, reinforcing the contribution of biochemical markers in disease characterization.

Collectively, these findings demonstrate that while traditional diagnostic criteria effectively identify hallmark symptoms, they fail to capture subtle clinical and biochemical variations detectable by ML approaches. Thus, our proposed ML framework is designed not to replace but to complement the ICBD criteria, offering a more comprehensive and data‐driven diagnostic perspective that enhances clinical decision‐making and supports future integration into real‐world clinical workflows.

## 7. Study Limitations and Future Work

### 7.1. Challenges in Translating the Proposed ML Models Into Clinical Practice

Even though the suggested ML models perform well, there are a number of obstacles to overcome before they can be used in actual clinical settings. It should be emphasized that the current study was not intended to provide an autonomous diagnostic system but rather to evaluate the viability and potential of ML tools in supporting clinicians and facilitating diagnostic reasoning. First and foremost, strong interoperability standards and safe data exchange mechanisms are necessary for the integration of ML tools into current hospital information systems. Second, clinical data is frequently noisy, incomplete, and heterogeneous, which can impact model reliability when used outside of a controlled study environment. Third, doctors need a clear explanation for every prediction in order to gain trust, so model interpretability and transparency are critical to their acceptance of ML‐based recommendations. Other obstacles include ethical and legal issues like patient data privacy, liability for incorrect classification, and the requirement for ongoing model performance monitoring. To guarantee the safe and efficient integration of ML models into routine clinical procedures, these issues must be resolved for the translation of ML models into daily clinical workflows.

### 7.2. Limitations

There are some restrictions on this study. Generalizability may be limited by the small dataset size and the fact that it came from a single institution. Furthermore, we acknowledge that external validation on separate datasets is a crucial next step, but it was not carried out. Notwithstanding these drawbacks, the reliability of our results is supported by consistent performance across several models and feature‐importance techniques. Nevertheless, because this study was exploratory in nature, its main goal was to generate insights and assess the usefulness of ML techniques in enhancing diagnostic understanding of BD, not to create a finalized clinical diagnostic tool.

### 7.3. Roadmap for Clinical Implementation

We offer a methodical road map for getting from research to clinical adoption. To verify generalizability across various populations, the models are first validated on external, multicenter datasets. Following validation, the models can be incorporated as a clinical decision‐support tool into hospital electronic medical record (EMR) systems. Instead of replacing clinical expertise at this point, ML systems should continue to function as knowledge‐driven, assistive tools that improve clinicians’ decision‐making. To guarantee seamless integration and adherence to data protection laws, clinicians, data scientists, and IT departments must work closely together. In order to improve trust and usability, training programs should be created to assist clinicians in interpreting model outputs, particularly those that are based on SHAP. It is also necessary to set up ongoing postdeployment monitoring in order to identify performance drift and guarantee accuracy over time. In the end, ethical supervision and regulatory approval will be crucial steps prior to widespread clinical adoption. This roadmap, which outlines a future path where ML can enhance diagnostic reasoning and broaden clinical understanding rather than replace physician diagnostics, thus reflects the exploratory and translational nature of the current work.

## 8. Conclusions

BD is considered an immune‐rheumatic disease that is difficult to diagnose, and much research has appeared examining and discussing diagnostic criteria. Moreover, we found no published research examining the diagnosis of BD using ML models with clinical data. In this paper, we classify BD using five models: DT, bagging, RF, XGBoost, and SVM. RF achieved the best performance with an accuracy of 97% and an AUC of 1, followed by XGBoost with comparable performance. This study was designed as an exploratory and knowledge‐oriented investigation aimed at assessing the feasibility and potential of ML techniques in facilitating the diagnostic process of BD, rather than replacing clinical judgment or automating the diagnostic decision. In conclusion, this is an exploratory study of the effectiveness of ML models in diagnosing BD. ML models are somewhat ambiguous and do not provide predictive factors. This study resolved ambiguity by identifying factors closely associated with diagnosis through three distinct methods: inherent feature importance, permutation‐based analysis, and SHAP values. Our findings highlight arthritis, followed by oral ulcers, albumin, and anti‐DNA, which significantly impact the model as highly relevant features, alongside several others such as PT, ALT, CRP, creatinine, and ESR. These findings demonstrate the capability of ML models to enhance diagnostic understanding and support physicians by uncovering clinically meaningful patterns rather than serving as independent diagnostic systems. This insight aids physicians in pinpointing the most crucial disease‐detecting features and making more informed clinical decisions. Future work will remain exploratory in nature, focusing on validating the generalizability and practical applicability of ML‐assisted approaches across multicenter datasets and diverse clinical settings. Such a study will not only validate the generalizability of our results but also enable clinical translation.

## Ethics Statement

The study was conducted in accordance with approval by the Ethics Committee at King Abdulaziz University Hospital in Jeddah, Saudi Arabia, IRB No. (HA‐02‐J‐008), and date Monday (April 22, 2024).

## Disclosure

All authors have reviewed the published version of the manuscript.

## Conflicts of Interest

The authors declare no conflicts of interest.

## Author Contributions

Conceptualization: H.B.A., N.A., A.A., H.B., and S.A. Proposed method: H.B.A. and H.B. Data manipulation: H.B.A. and Y.B. Software and implementation: H.B.A. Data analysis: H.B.A. Validation: H.B.A. Writing and preparing drafts: H.B.A. and H.A. Preparing figures and tables: H.B.A. Follow‐up on work implementation: N.A. Supervision: N.A. and S.A. Clinical data collection: Y.B. and M.M. Obtain ethical approval: H.B.A., A.A., and Y.B.

## Funding

The study was funded by the Deanship of Scientific Research (DSR) at King Abdulaziz University, Jeddah (1029‐612‐2024).

## Supporting information


**Supporting Information** Additional supporting information can be found online in the Supporting Information section. Table S1. Definition and clinical relevance of the demographic, clinical, and laboratory features included in the Behçet’s disease dataset. Table S2. Descriptive statistics and data types for all 42 features, including summary values and completeness information. Table S3. Value mapping and categorical encodings are applied to clinical and laboratory features during preprocessing. Table S4. Frequency distributions and missing data summary for each feature in the dataset. Table S5. Chi‐square test results showing the statistical associations between each feature and the diagnosis outcome.

## Data Availability

The complete dataset used in this study is available, and interested researchers can request access to the data directly from the authors.
