# Supplementary material for: Enhancing the Diagnosis of Behçet’s Disease Using Machine Learning: A Comparative Study on Clinical Data From Saudi Arabia
Source: Int J Telemed Appl. 2026 Jan 24;2026:6157852. doi: 10.1155/ijta/6157852 (PMC12831129; doi:10.1155/ijta/6157852)
Supplement: Supplementary file 1 — Supporting Information Additional supporting information can be found online in the Supporting Information section. Table S1. Definition and clinical relevance of the demographic, clinical, and laboratory features included in the Behçet’s disease dataset. Table S2. Descriptive statistics and data types for all 42 features, including summary values and completeness information. Table S3. Value mapping and categorical encodings are applied to clinical and laboratory features during preprocessing. Table S4. Frequency distributions and missing data summary for each feature in the dataset. Table S5. Chi‐square test results showing the statistical associations between each feature and the diagnosis outcome. [file IJTA-2026-6157852-s001.docx]

**Appendix: BD** **Dataset Details**

**Table A1-Definition and clinical relevance of features included in the Behçet’s disease dataset.**

detailed definitions and clinical justifications for the 42 demographic, clinical, and laboratory features used in the machine learning models for Behçet’s disease diagnosis. The relevance of each feature is explained based on its diagnostic or pathophysiological significance.

| **Definition** | **Name of the Feature** |  |
| --- | --- | --- |
| relevant because Behçet’s disease commonly presents in young adulthood, and younger onset is linked to more severe manifestations. | Age | 1 |
| relevant because Behçet’s disease prevalence and severity differ across populations, being more common along the Silk Road regions. | NATIONALITY | 2 |
| relevant as the most frequent and primary diagnostic feature of Behçet’s disease | Oral Ulcers | 3 |
| relevant because recurrent genital ulcers are a key diagnostic criterion for Behçet’s disease and are highly specific compared to other features. | Genital Ulcers | 4 |
| relevant because uveitis and other ocular involvements are major diagnostic criteria in Behçet’s disease and strongly associated with risk of vision loss. | Ocular Lesions | 5 |
| relevant because erythema nodosum, pseudofolliculitis, and acneiform lesions are common manifestations of Behçet’s disease and contribute to diagnosis. | Skin Lesions | 6 |
| relevant because central nervous system involvement in Behçet’s disease can lead to severe complications, including meningoencephalitis, cerebral venous thrombosis, and long-term neurological deficits. | Neurological Manifestations | 7 |
| relevant because joint involvement in Behçet’s disease is usually non-erosive and affects peripheral joints, contributing to disease assessment but not a major diagnostic criterion. | Arthritis | 8 |
| relevant because it is a common skin manifestation of Behçet’s disease, often presenting as tender red nodules, and supports the clinical diagnosis. | Erythema Nodosum | 9 |
| relevant because inflammation of superficial veins is a common vascular manifestation in Behçet’s disease and aids in supporting the diagnosis. | Superficial Phlebitis | 10 |
| relevant because DVT is a serious vascular complication of Behçet’s disease, reflecting involvement of large veins and contributing to morbidity. | Deep Vein Thrombosis  (DVT) | 11 |
| relevant because thrombosis of major veins (e.g., vena cava, hepatic veins) is a severe vascular manifestation of Behçet’s disease and may lead to significant complications. | Large Vein Thrombosis | 12 |
| relevant because arterial involvement in Behçet’s disease, though less common than venous, can lead to serious complications such as aneurysms, ischemia, or organ damage. | Arterial Thrombosis | 13 |
| relevant because it is a frequent ocular manifestation of Behçet’s disease, potentially causing vision loss and included as a major diagnostic criterion. | Uveitis | 14 |
| relevant because inflammation of retinal vessels is a severe ocular manifestation of Behçet’s disease, associated with vision impairment and included in disease assessment. | Retinal Vasculitis | 15 |
| relevant because a skin hypersensitivity reaction to minor trauma is a supportive diagnostic feature of Behçet’s disease, reflecting hyper-reactivity of the immune system. | Positive Pathergy Test | 16 |
| relevant because elevated ESR indicates systemic inflammation in Behçet’s disease, helping to monitor disease activity, though it is non-specific. | Erythrocyte Sedimentation  Rate (ESR) | 17 |
| relevant because elevated CRP reflects systemic inflammation in Behçet’s disease and can be used to monitor disease activity, although it is non-specific. | C-reactive protein (CRP) | 18 |
| relevant because anemia may occur in Behçet’s disease due to chronic inflammation or gastrointestinal involvement. | Hemoglobin (Hb) | 19 |
| relevant because thrombocytosis or abnormal platelet counts may reflect systemic inflammation or vascular involvement in Behçet’s disease. | platelet count (PLT) | 20 |
| relevant because leukocytosis may occur in Behçet’s disease during active inflammation, helping to monitor disease activity, though it is non-specific. | white blood count (WBC) | 21 |
| relevant because elevated AST may indicate liver involvement. | Aspartate Transferase (AST) | 22 |
| relevant because elevated ALT may indicate liver involvement. | Alanine Aminotransferase  (ALT) | 23 |
| relevant because elevated bilirubin may indicate liver dysfunction or hemolysis in patients with Behçet’s disease, although it is a non-specific marker. | Bilirubin | 24 |
| relevant because low serum albumin may reflect chronic inflammation, malnutrition, or protein loss in patients with Behçet’s disease. | Albumin | 25 |
| relevant because RF is usually negative in Behçet’s disease, helping to differentiate it from rheumatoid arthritis and other autoimmune disorders. | Rheumatoid Factor (RF) | 26 |
| relevant because anti-CCP antibodies are typically negative in Behçet’s disease, helping to distinguish it from rheumatoid arthritis. | Anti-cyclic citrullinated pep-  tide (anti-CCP) | 27 |
| relevant because PTT may be assessed to evaluate coagulation status in Behçet’s disease patients, especially those with vascular involvement or history of thrombosis. | Partial thromboplastin time  (PTT) | 28 |
| relevant because PT may be measured to assess coagulation status in Behçet’s disease patients, particularly in those with vascular involvement or thrombotic history. | Prothrombin time (PT) | 29 |
| relevant because INR is used to monitor coagulation status in Behçet’s disease patients, especially those on anticoagulant therapy for thrombotic complications. | International normalised ra-  tio (INR) | 30 |
| relevant because serum creatinine is used to assess kidney function in Behçet’s disease patients, particularly in those with renal involvement or on nephrotoxic medications. | Creatinine | 31 |
| relevant because ANA is usually negative in Behçet’s disease, helping to differentiate it from other autoimmune disorders such as systemic lupus erythematosus. | Antinuclear Antibodies (ANA) | 32 |
| relevant because anti-DNA antibodies are typically negative in Behçet’s disease, helping to distinguish it from systemic lupus erythematosus and other autoimmune disorders. | Anti DNA | 33 |
| relevant because anti-SSA antibodies are usually negative in Behçet’s disease, helping to differentiate it from Sjögren’s syndrome and other autoimmune disorders. | Anti-Sjögren’s syndrome A  (anti SSA) | 34 |
| relevant because SCL-70 antibodies are typically negative in Behçet’s disease, helping to differentiate it from systemic sclerosis and related autoimmune disorders. | Scleroderma (SCL-70) | 35 |
| relevant because ANCA are usually negative in Behçet’s disease, helping to differentiate it from ANCA-associated vasculitides. | Antineutrophil Cytoplasmic  Antibodies (ANCA) | 36 |
| relevant because these antibodies are usually negative in Behçet’s disease, helping to distinguish it from antiphospholipid syndrome and other thrombotic disorders. | Anti Cardiolipin | 37 |
| relevant because HCV infection should be screened in Behçet’s disease patients, before initiating immunosuppressive therapy. | hepatitis C (HCV) | 38 |
| relevant because HBV infection should be screened in Behçet’s disease patients before immunosuppressive therapy. | Hepatitis B (HBV) | 39 |
| relevant because echocardiography may detect cardiac involvement (e.g., endocarditis, pericarditis, intracardiac thrombi) in Behçet’s disease, though it is not a primary diagnostic criterion. | ECHO | 40 |
| relevant because it reflects glycemic control and is not specific to Behçet’s disease, but may be important. | HBA1C | 41 |
| relevant because lipid assessment is not diagnostic for Behçet’s disease but could be an indicator, especially in patients with vascular involvement. | LDL | 42 |

**Table A2-Descriptive statistics and data type summary of all features in the Behçet’s disease dataset.**

This table presents an overview of the 42 demographic, clinical, and laboratory features included in the Behçet’s disease dataset. It provides summary statistics (average, minimum, and maximum values), the number of missing and non-missing cases, and the data type for each feature. These descriptive metrics help illustrate data completeness, variability, and distribution patterns across the patient cohort (n = 148).

| Example | Average | Max | Min | Missing  N | Non-Missing Patients | Type | Feature Name |  |
| --- | --- | --- | --- | --- | --- | --- | --- | --- |
| Demographics | | | | | | | | |
| 4,10,80,… | 43.56 | 80 | 4 | 1 | 147 | Continuous | Age | 1 |
| SA, AF, PALESTINIAN… |  |  |  | 0 | 148 | text | NATIONALITY | 2 |
| Clinical symptoms | | | | | | | | |
| Yes, No |  |  |  | 0 | 148 | Categorical | Oral Ulcers | 3 |
| Yes, No |  |  |  | 0 | 148 | Categorical | Genital Ulcers | 4 |
| Yes, No |  |  |  | 0 | 148 | Categorical | Ocular Lesions | 5 |
| Yes, No |  |  |  | 0 | 148 | Categorical | Skin Lesions | 6 |
| Yes, No |  |  |  | 0 | 148 | Categorical | Neurological Manifestations | 7 |
| Yes, No |  |  |  | 0 | 148 | Categorical | Arthritis | 8 |
| Yes, No |  |  |  | 0 | 148 | Categorical | Erythema Nodosum | 9 |
| Yes, No |  |  |  | 0 | 148 | Categorical | Superficial Phlebitis | 10 |
| Yes, No |  |  |  | 0 | 148 | Categorical | Deep Vein Thrombosis (DVT) | 11 |
| Yes, No |  |  |  | 0 | 148 | Categorical | Large Vein Thrombosis | 12 |
| Yes, No |  |  |  | 0 | 148 | Categorical | Arterial Thrombosis | 13 |
| Yes, No |  |  |  | 0 | 148 | Categorical | Uveitis | 14 |
| Yes, No |  |  |  | 0 | 148 | Categorical | Retinal Vasculitis | 15 |
| Yes, No |  |  |  | 0 | 148 | Categorical | Positive Pathergy Test | 16 |
| Laboratory test data | | | | | | | | |
|  | 20.79 | 102 | 1 | 34 | 114 | Continuous | Erythrocyte Sedimentation  Rate (ESR) | 17 |
|  | 12.17 | 184 | 3.3 | 21 | 127 | Continuous | C-reactive protein (CRP) | 18 |
|  | 12.73 | 18.1 | 5.32 | 6 | 142 | Continuous | Hemoglobin (Hb) | 19 |
|  | 296.36 | 669 | 70 | 6 | 142 | Continuous | platelet count (PLT) | 20 |
|  | 7.83 | 81 | 2.6 | 6 | 142 | Continuous | white blood count (WBC) | 21 |
|  | 26.56 | 152 | 6 | 19 | 129 | Continuous | Aspartate Transferase (AST) | 22 |
|  | 48.24 | 982 | 7 | 19 | 129 | Continuous | Alanine Aminotransferase  (ALT) | 23 |
|  | 11.04 | 222 | 2 | 19 | 129 | Continuous | Bilirubin | 24 |
|  | 37.63 | 51.6 | 28.5 | 20 | 128 | Continuous | Albumin | 25 |
| Positive, Negative |  |  |  | 1 | 147 | Categories | RF | 26 |
| Positive, Negative |  |  |  | 1 | 147 | Categories | anti-CCP | 27 |
| Abnormal, Normal |  |  |  | 19 | 129 | Categories | PTT | 28 |
| Abnormal, Normal |  |  |  | 18 | 130 | Categories | PT | 29 |
| Abnormal, Normal |  |  |  | 20 | 128 | Categories | INR | 30 |
|  | 69.05 | 310 | 5 | 21 | 127 | Continuous | Creatinine | 31 |
| Positive, Negative |  |  |  | 35 | 113 | Categories | ANA | 32 |
| Positive, Negative |  |  |  | 2 | 146 | Categories | Anti DNA | 33 |
| Positive, Negative |  |  |  | 2 | 146 | Categories | anti SSA | 34 |
| Positive, Negative |  |  |  | 1 | 147 | Categories | SCL-70 | 35 |
| Positive, Negative |  |  |  | 1 | 147 | Categories | ANCA | 36 |
| Positive, Negative |  |  |  | 3 | 145 | Categories | Anti Cardiolipin | 37 |
| Positive, Negative |  |  |  | 1 | 147 | Categories | HCV | 38 |
| Positive, Negative |  |  |  | 1 | 147 | Categories | HBV | 39 |
| very low, Mildly decreased,  Normal |  |  |  | 111 | 37 | Categories | ECHO | 40 |
| Abnormal, Normal |  |  |  | 77 | 71 | Categories | HBA1C | 41 |
| Abnormal, Normal |  |  |  | 49 | 99 |  | LDL | 42 |

Delete Columns that contain missing data more than 25% for example ECHO EF%, HBA1C,LDL

**Table A3-Value mapping and categorical encoding of clinical and laboratory features in the Behçet’s disease dataset.**

This table outlines the categorical and numerical value mappings applied during the preprocessing phase. Continuous laboratory variables were discretized into clinically meaningful ranges, while binary and categorical clinical features (e.g., Yes/No, Positive/Negative, Normal/Abnormal) were standardized for consistency across the dataset. These encodings ensured uniform representation of the 42 features for model training and analysis.

| Value | Term | Feature |
| --- | --- | --- |
|  |  |  |
| ≤20 years  21-30 years  >30 years | young  middle  old | Age |
| SA  Non-SA | Saudi  non-Saudi | NATIONALITY |
|  | Yes  No | Oral Ulcers |
|  | Yes  No | Genital Ulcers |
|  | Yes  No | Ocular Lesions |
|  | Yes  No | Skin Lesions |
|  | Yes  No | Neurological Manifestations |
|  | Yes  No | Arthritis |
|  | Yes  No | Erythema Nodosum |
|  | Yes  No | Superficial Phlebitis |
|  | Yes  No | DVT |
|  | Yes  No | Large Vein Thrombosis |
|  | Yes  No | Arterial Thrombosis |
|  | Yes  No | Uveitis |
|  | Yes  No | Retinal Vasculitis |
|  | Yes  No | Positive Pathergy Test |
| ≥ 20 U/mL  < 20 U/mL | Positive  Negative | RF |
| ≥ 20 U/mL  < 20 U/mL | Positive  Negative | anti-CCP |
| 20 – 40 Seconds  other | Normal  Abnormal | PTT |
| 11-14 seconds  other | Normal  Abnormal | PT |
| 0.9 - 1.2 U/mL  other | Normal  Abnormal | INR |
| if titer 1.40 or mor  other | Positive  Negative | ANA |
| ≥ 30 U/mL  < 30 U/mL | Positive  Negative | Anti DNA |
| ≥ 20 U/mL  < 20 U/mL | Positive  Negative | anti SSA |
| ≥ 20 U/mL  < 20 U/mL | Positive  Negative | SCL-70 |
| ≥ 20 U/mL  < 20 U/mL | Positive  Negative | ANCA |
| ≥ 15 U/mL  < 15 U/mL | Positive  Negative | Anti Cardiolipin |
|  | Positive  Negative | HCV |
|  | Positive  Negative | HBV |

**Table A4-Frequency distribution and missing data summary for each feature in the Behçet’s disease dataset.**

This table presents the frequency of each feature’s subcategories and the number of missing entries within the dataset. It provides an overview of data completeness and the prevalence of clinical and laboratory findings among the 148 patients (76 diagnosed with Behçet’s disease and 72 with rheumatoid arthritis), forming the basis for subsequent machine learning experiments.

| Missing | Frequency | Subcategory | Feature |
| --- | --- | --- | --- |
| 1 | 6 | ≤20 | Age |
| 1 | 19 | (21-30) |  |
| 1 | 122 | (>30) |  |
| 0 | 28 | SA | Nationality |
| 0 | 120 | not SA |  |
| 0 | 107 | NO | Oral ulcers |
| 0 | 41 | YES |  |
| 0 | 121 | NO | Genital Ulcers |
| 0 | 27 | YES |  |
| 0 | 147 | NO | Ocular Lesions |
| 0 | 1 | YES |  |
| 0 | 139 | NO | Skin Lesions |
| 0 | 9 | YES |  |
| 0 | 147 | NO | Neurological Manifestations |
| 0 | 1 | YES |  |
| 0 | 59 | NO | Arthritis |
| 0 | 89 | YES |  |
| 0 | 148 | NO | Erythema Nodosum |
| 0 | 0 | YES |  |
| 0 | 148 | NO | Superficial Phlebitis |
| 0 | 0 | YES |  |
| 0 | 139 | NO | Deep Vein Thrombosis (DVT) |
| 0 | 9 | YES |  |
| 0 | 146 | NO | Large Vein Thrombosis |
| 0 | 2 | YES |  |
| 0 | 147 | NO | Arterial Thrombosis |
| 0 | 1 | YES |  |
| 0 | 131 | NO | Uveitis |
| 0 | 17 | YES |  |
| 0 | 147 | NO | Retinal Vasculitis |
| 0 | 1 | YES |  |
| 0 | 146 | NO | Positive Pathergy Test |
| 0 | 2 | YES |  |
| 1 | 27 | Positive | RF |
| 1 | 120 | Negative |  |
| 1 | 18 | Positive | ANTICCP |
| 1 | 129 | Negative |  |
| 19 | 10 | Abnormal | PTT |
| 19 | 119 | Normal |  |
| 18 | 24 | Abnormal | PT |
| 18 | 106 | Normal |  |
| 20 | 12 | Abnormal | INR |
| 20 | 116 | Normal |  |
| 35 | 60 | Negative | ANA |
| 35 | 53 | Positive |  |
| 2 | 25 | Positive | AntidDNA |
| 2 | 121 | Negative |  |
| 2 | 1 | Positive | AntiSSA |
| 2 | 145 | Negative |  |
| 1 | 147 | Negative | AntiSCL_70 |
| 1 | 0 | Positive |  |
| 1 | 4 | Positive | ANCA |
| 1 | 143 | Negative |  |
| 3 | 3 | Positive | Anti_Cardiolipin |
| 3 | 142 | Negative |  |
| 1 | 3 | Positive | HCV |
| 1 | 144 | Negative |  |
| 1 | 147 | Negative | HBV |
| 1 | 0 | Positive |  |
| 0 | 72 | Not Behcet’s | Diagnosis |
| 0 | 76 | Behcet’s |  |

**Table A5 -Chi-square test results showing the statistical association between each feature and Behçet’s disease diagnosis.**

This table presents the results of the Chi-square (χ²) independence test performed between each clinical and laboratory feature and the diagnosis target (Behçet’s disease vs. rheumatoid arthritis). Features with p-values below 0.05 were considered statistically significant and thus potentially more discriminative for model training. Notably, oral ulcers, arthritis, RF, anti-CCP, and PT exhibited strong associations with Behçet’s disease (p < 0.001), supporting their clinical relevance and inclusion in subsequent feature selection analyses.

| **p-value** | **Chi2** | **Target** | **Feature** |
| --- | --- | --- | --- |
| 0.022 | 7٫612^a^ | Diagnosis | Age |
| 0.052 | 3٫766^a^ | Diagnosis | NATIONALITY |
| 0.000 | 53٫726^a^ | Diagnosis | Oral Ulcers |
| 0.000 | 31٫287^a^ | Diagnosis | Genital Ulcers |
| 0.329 | ٫954^a^ | Diagnosis | Ocular Lesions |
| 0.003 | 9٫078^a^ | Diagnosis | Skin Lesions |
| 0.329 | ٫954^a^ | Diagnosis | Neurological Manifestations |
| 0.000 | 92٫949^a^ | Diagnosis | Arthritis |
| 0.003 | 9٫078^a^ | Diagnosis | (DVT) |
| 0.166 | 1٫921^a^ | Diagnosis | Large Vein Thrombosis |
| 0.329 | ٫954^a^ | Diagnosis | Arterial Thrombosis |
| 0.000 | 18٫195^a^ | Diagnosis | Uveitis |
| 0.329 | ٫954^a^ | Diagnosis | Retinal Vasculitis |
| 0.166 | 1٫921^a^ | Diagnosis | Positive Pathergy Test |
| 0.007 | 102٫316^a^ | Diagnosis | (ESR) |
| 0.009 | 137٫356^a^ | Diagnosis | C-reactive protein (CRP) |
| 0.075 | 88٫823^a^ | Diagnosis | Hemoglobin (Hb) |
| 0.337 | 127٫985^a^ | Diagnosis | platelet count (PLT) |
| 0.324 | 143٫997^a^ | Diagnosis | white blood count (WBC) |
| 0.276 | 62٫890^a^ | Diagnosis | Aspartate Transferase (AST) |
| 0.220 | 88٫409^a^ | Diagnosis | (ALT) |
| 0.222 | 46٫508^a^ | Diagnosis | Bilirubin |
| 0.008 | 96٫539^a^ | Diagnosis | Albumin |
| 0.000 | 36٫452^a^ | Diagnosis | (RF) |
| 0.000 | 21٫631^a^ | Diagnosis | (anti-CCP) |
| 0.001 | 16٫995^a^ | Diagnosis | (PTT) |
| 0.000 | 48٫436^a^ | Diagnosis | (PT) |
| 0.000 | 27٫138^a^ | Diagnosis | INR |
| 0.386 | 95٫295^a^ | Diagnosis | Creatinine |
| 0.003 | 8٫684^a^ | Diagnosis | (ANA) |
| 0.000 | 33٫293^a^ | Diagnosis | Anti DNA |
| 0.592 | ٫288^a^ | Diagnosis | (anti SSA) |
| 0.037 | 4٫340^a^ | Diagnosis | (ANCA) |
| 0.694 | ٫155^a^ | Diagnosis | Anti Cardiolipin |
| 0.037 | 4٫340^a^ | Diagnosis | (HCV) |

**Appendix references**

[1] E. Kural-Seyahi, I. Fresko, N. Seyahi, Y. Ozyazgan, C. Mat, V. Hamuryudan, S. Yurdakul, and H. Yazici, “The long-term mortality and morbidity of Behçet syndrome: a 2-decade outcome survey of 387 patients followed at a dedicated center,” Medicine (Baltimore), vol. 82, no. 1, pp. 60–76, 2003.

[2] International Team for the Revision of the International Criteria for Behçet’s Disease (ICBD), “The International Criteria for Behçet’s Disease (ICBD): a collaborative study of 27 countries on the sensitivity and specificity of the new criteria,” J. Eur. Acad. Dermatol. Venereol., vol. 28, no. 3, pp. 338–347, 2014.

[3] E. Seyahi, “Behçet’s disease: How to diagnose and treat vascular involvement,” Best Pract. Res. Clin. Rheumatol., vol. 30, no. 2, pp. 279–295, 2016.

[4] H. Yazici, E. Seyahi, G. Hatemi, and Y. Yazici, “Behçet syndrome: a contemporary view,” Nat. Rev. Rheumatol., vol. 14, no. 2, pp. 107–119, 2018.

[5] G. Hatemi, E. Seyahi, I. Fresko, and H. Yazici, “Management of Behçet’s syndrome,” Rheumatology (Oxford), vol. 57, suppl. 1, pp. i61–i70, 2018.
